# Antioxidant action and cytotoxicity on HeLa and NIH-3T3 cells of new quercetin derivatives

**DOI:** 10.2478/intox-2013-0031

**Published:** 2013-12

**Authors:** Martina Danihelová, Miroslav Veverka, Ernest Šturdík, Soňa Jantová

**Affiliations:** 1 Institute of Biochemistry, Nutrition and Health Protection, Faculty of Chemical and Food Technology, Slovak University of Technology, Bratislava, Slovakia; 2 Eurofins Bel/Novamann s.r.o., Nové Zámky, Slovakia

**Keywords:** antioxidant, cytotoxicity, HeLa, NIH-3T3, quercetin derivatives

## Abstract

Quercetin is a natural polyphenol with proven health beneficial activities. In this study 15 new quercetin derivatives were prepared with the aim to enhance their bioavailability. Modification of their physicochemical properties could herewith improve the action in cells. The prepared compounds were tested for their antioxidant and cytotoxic activity. The ability to scavenge free radicals as well as ferric reducing antioxidant power of the new derivatives was not better than that of unmodified quercetin. But for acetylated esters a better cytotoxic activity was found on human cervical cancer cells HeLa than for the initial molecule. The best effect revealed chloronaphtoquinone quercetin (IC_50_=13.2 µM). For this compound comparable cytotoxic action on non-cancer murine fibroblast cells was detected (IC_50_=16.5 µM). The obtained results indicate that appropriate lipophilization of the quercetin molecule could improve its cytotoxic action in cells, probably due to its enhanced bioavailability.

## Introduction

Cancer diseases represent with their contribution of 20 to 25% the second leading cause of death in the world. In 2007, 28 131 new cancer cases were registered in Slovakia. The incidence and mortality of cancer in recent years in the Slovak population is displaying a raising trend. For this rapid increase and large occurrence mainly prostate and colorectal cancers are responsible in males. In females the dramatic increase in cancer cases was caused by breast, female genital organs and lung cancers (Diba *et al.*, 2012). It is therefore important to deal with the study of new potential anticancer drugs that are effective in selective inhibition of cancer cell growth.

Flavonoids represent such a group of natural, low toxic compounds. They are polyphenolics abundant in the plant kingdom. The average human diet contains a considerable amount of flavonoids and the major dietary sources are fruits (apple, orange, strawberry, grapes), vegetables (onion, broccoli, parsley, cabbage), soybeans and different herbs (sage, mint, tea leaves, oregano). Flavonoids have for decades been known for their broad spectrum of identified biological activities. As a part of medicinal herbs they were used for treatment purposes from ancient times. Later experiments and studies have shown that they dispose of many health beneficial properties, e.g. anti-inflammatory, cardioprotective, anti-allergic or hepatoprotective effects (Xiao *et al.*, 2011). In recent years there has been a growing interest in studying their anticancer effects (Moghaddam *et al.*, 2012; Moheb *et al.*, 2013).

Published articles have revealed that flavonoids are able to inhibit the growth of cancer cells *in vitro* (Delmulle *et al.*, 2006; Chidambara Murthy *et al.*, 2012). These findings are confirmed by several *in vivo* studies (Batra and Sharma, 2013). Flavonoids exert their anticancer action through affecting key mechanisms involved in cancer pathogenesis. They are effective antioxidant and anti-inflammatory agents. In initial stages, they inhibit metabolic activation of carcinogens. In progression phases they induce apoptosis, inhibit angiogenesis, cancer cell proliferation and tumor metastasis, and they also modulate multidrug resistance (Clere *et al.*, 2011).

Among flavonoids, quercetin is considered an excellent free radical scavenging antioxidant. Apart from this quercetin also exerts a direct pro-apoptotic effect on tumor cells and can indeed block the growth of several cancer cell lines at different phases of the cell cycle. Both these effects have been documented in a wide variety of cellular models as well as in animal models (Gibellini *et al.*, 2011). Several studies demonstrated a significant role of quercetin in growth inhibition of breast, colon, prostate, ovary, endometrium and lung cancer cells (Baghel *et al.*, 2012). But its effect is highly dependent on the ability to penetrate the lipophilic bilayer of the cell membranes.

Literature documents that the bioavailability of quercetin is not sufficient (Viskupicová *et al.*, 2008). Authors tried to overcome this disadvantage via modification of the quercetin structure with groups of various polarities. According to published data, it can be concluded that improvement of anticancer properties was seen mainly upon flavonoid structure lipophilization (Cárdenas *et al.*, 2006; Salem *et al.*, 2011).

In this study we tested bioactivities of 15 novel quercetin derivatives with various acyl donors. We assessed their *in vitro* antioxidant and cytotoxic activity. The relationship between the observed action and polarity of molecules as well as the determined activities were also evaluated.

## Material and methods

### Material

Dulbecco's modified eagle medium (DMEM), adult bovine serum (BOS), penicillin-streptomycin solution, thiazolyl blue tetrazolium bromide, 2,2’-diphenyl-1-picrylhydrazyl (DPPH), 2,4,6-tris(2-pyridyl)-S-triazine (TPTZ) and dimethyl sulfoxide (DMSO) for HPLC were purchased from Sigma-Aldrich (DE). All other solvents and reagents were supplied from local companies (Mikrochem, SK; Vitrum VWR, CZ) and were of analytical grade.

### Quercetin derivatives

Quercetin structure was modified using simple condensation reactions or selective protection procedures and subsequent acylation with acylchlorides (Veverka *et al.*, 2013). The structure of the compounds tested is outlined in Table 1. The purity of all the samples tested as determined by HPLC was approximately 99.8%.

**Table 1:** Chemical structure of quercetin derivatives.

No.	Name	Formula	Molecular weight
**1**	diquercetin	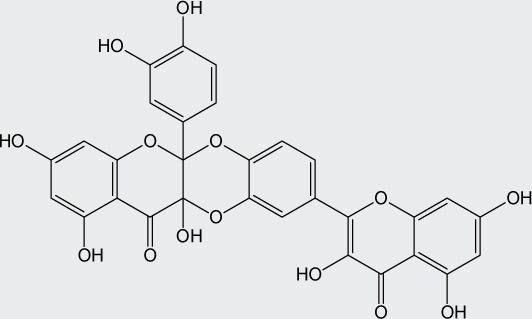	602.46
**2**	monochloropivaloyl quercetin	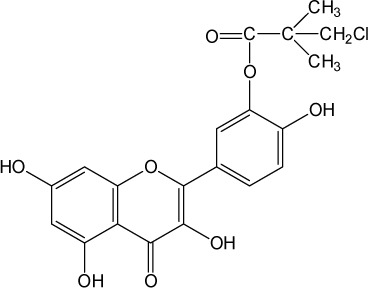	420.80
**3**	tri(monochloropivaloyl) quercetin	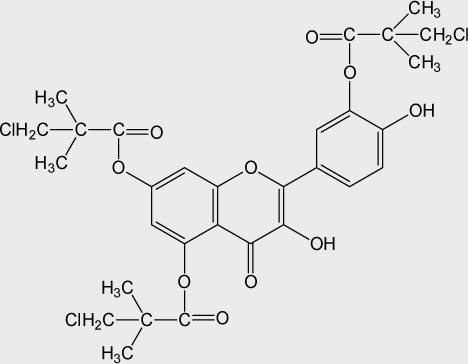	657.92
**4**	5-morfolinohydroxypropoxy quercetin	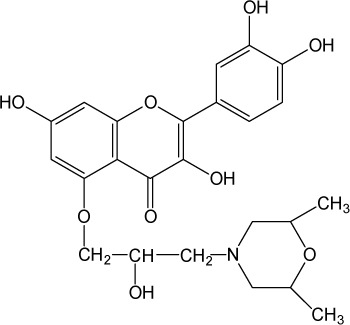	473.47
**5**	chloronaphtoquinone quercetin	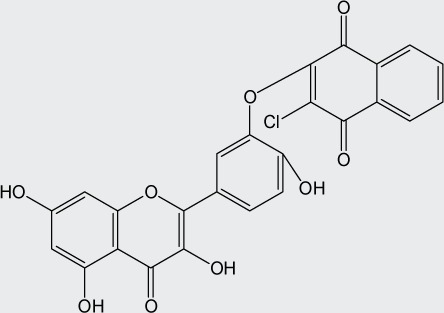	492.82
**6**	pentaacetyl quercetin	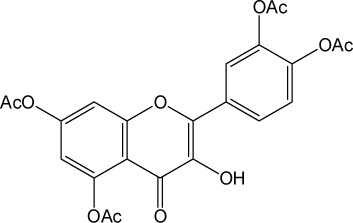	512.42
**7**	tri(diprenylcaffeoyl) quercetin	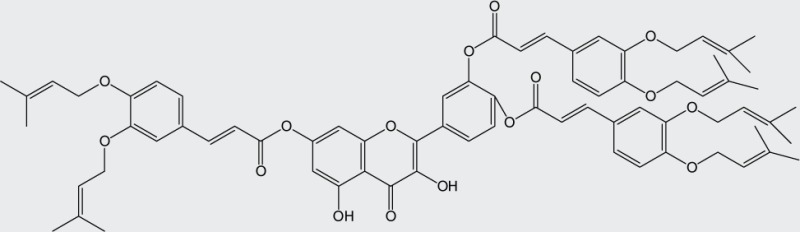	1196.50
**8**	di(diprenylcaffeoyl) quercetin	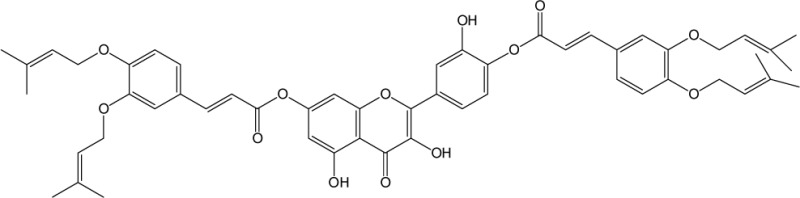	899.00
**9**	di(tetraacetylquinoyl) quercetin	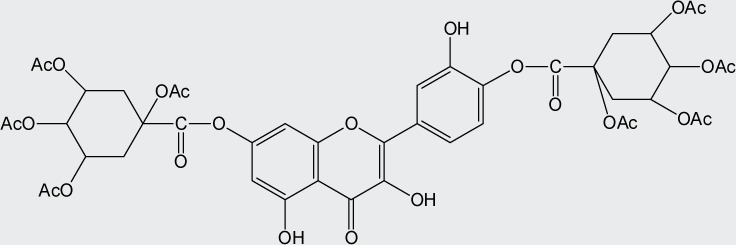	986.20
**10**	tri(trimethylgalloyl) quercetin	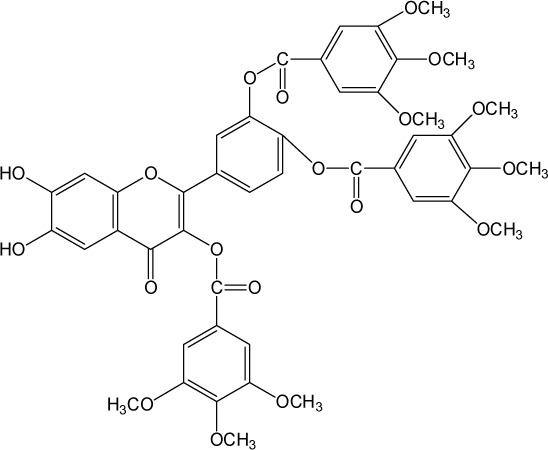	884.79
**11**	tri(diacetylcaffeoyl) quercetin	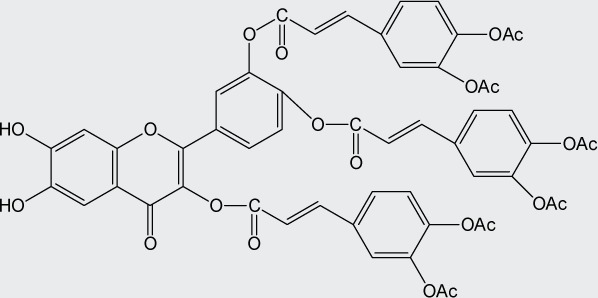	1040.90
**12**	tetra(acetylsalicyloyl) quercetin	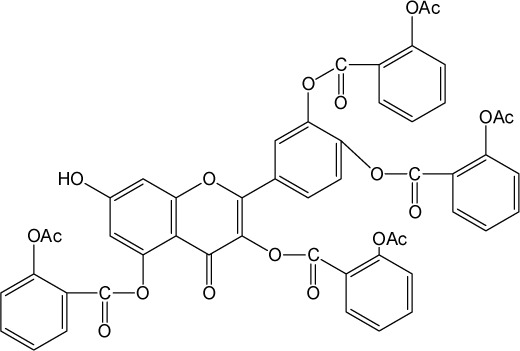	950.80
**13**	tri(acetylferuloyl) quercetin	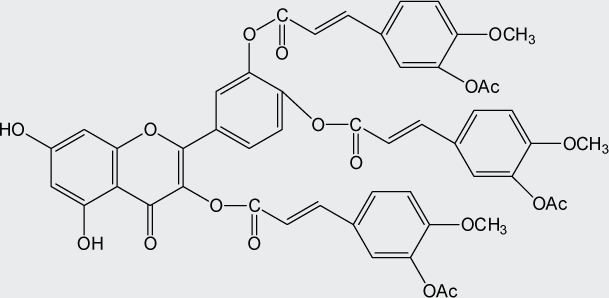	956.85
**14**	di(prenylferuloyl) quercetin	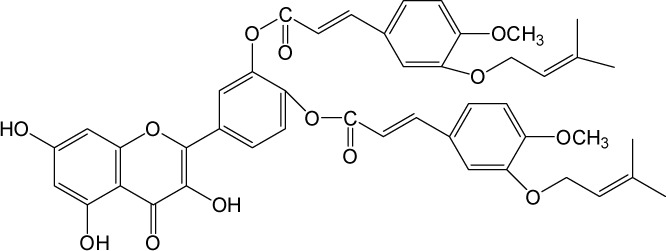	790.82
**15**	monoacetylferuloyl quercetin	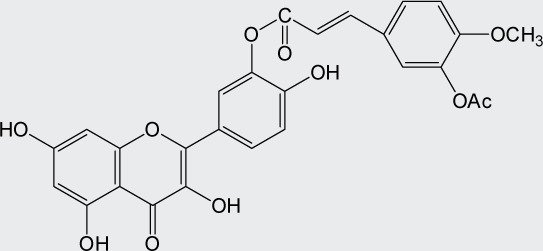	520.45

### Cell lines

The human tumor cell line HeLa and murine fibroblast NIH-3T3 cells were obtained from the ATCC (Rockville, MD, USA). The cells grown at 37 °C in humidified 5%-CO_2_ and 95%-air atmosphere were in Dulbecco's modified eagle medium supplemented with 10% (vol/vol) inactivated bovine serum, penicillin G and streptomycin (100 mg/l). Before a uniform monolayer of cells was formed, cells were freed from the surface of the culture flask by a 0.25% solution of trypsin and were subcultivated two to three times a week. The cells were plated on the cultivation flask (surface 25 cm^2^) at a density of 4×10^4^ HeLa cells and 6×10^4^ NIH-3T3 cells per ml medium and incubated for 24 h prior to the experiments.

### Scavenging of DPPH radical

We used the simple spectrophotometric method according to Yen and Chen (1995) to determine the ability to scavenge free radicals. Binding of DPPH? radical by compounds causes its decolorization. The compounds tested were diluted in methanol to the final concentration 200 µM to 12.5 µM. Trolox served as standard antioxidant control. Reactions of samples with DPPH? solution were performed in 96-well plates and lasted for 10 min at room temperature in the dark. After that, the absorbance changes were measured spectrophotometrically at 520 nm (Multiskan FC, Thermo Scientific, DE). Each value is the mean of 5 wells with standard deviation. Inhibitory concentrations (IC_50_) were expressed as the concentration of the derivative tested to react with one half of the DPPH? and were calculated by SigmaPlot.

### Ferric reducing antioxidant power (FRAP)

The antioxidant reducing power of the derivatives tested was performed using the FRAP method according to Benzie and Strain (1996). Ferric to ferrous ion reduction at low pH causes the formation of a colored ferrous-tripyridyltriazine complex. Samples diluted in methanol (200 µM to 12.5 µM) and FRAP reagent reacted for 10 min at 37 °C in 96-well microplates. At the end, absorbance changes were measured spectrophotometrically at 593 nm (Multiskan FC, Thermo Scientific, DE). Each value is the mean of 5 wells with standard deviation. Inhibitory concentrations (IC_50_) were expressed as the concentration of the derivative tested reducing one half of ferric complexes and were calculated by SigmaPlot.

### MTT assay

The cytotoxic effect of the compounds prepared on cells was detected *in vitro* using the mitochondrial cytotoxic test according to Jantová *et al.* (2010) with modifications. Cell viability was evaluated using thiazolyl blue tetrazolium bromide (MTT), which indicates the metabolic activity of cells. The experiment was performed in 96-well microplates. The cells were seeded at a density of 3.5×10^3^ HeLa or NIH-3T3 cells per well. Samples were dissolved in DMSO (stock solution 10 mM) and subsequently diluted in medium to the final concentration of 5 µM to 100 µM (concentration of DMSO 0.5%) and after 24 h they were added to the cells. Microplates were cultivated for 72 h in thermostat at 37 °C and 5% CO_2_ atmosphere. After incubation thiazolyl blue tetrazolium bromide (3.33 mg/ml phosphate buffered saline, pH=7.4) was pipetted to each well and left to incubate for further two hours. Then the medium with MTT solution was removed. Formazan crystals in viable cells were dissolved in the lysis solution (4 mM HCl and 0.1% Nonidet P40 in ethanol). Microplates were shaken 15 min at 1500 rpm. Absorbance was measured at 540 nm and reference wavelength at 740 nm (Multiskan FC, Thermo Scientific, DE). Each value is the mean of 6 wells with standard deviation. Inhibition activity was expressed as percentages of control with DMSO. For each compound IC_50_ values were calculated by SigmaPlot.

## Results

We chose two simple spectrophotometric methods to investigate antioxidant properties of the prepared quercetin derivatives. As reference standard synthetic analogue of vitamin E (trolox) was used. The 50% effective doses of samples (IC_50_) are shown in Table 2.

**Table 2:** Antioxidant activity of quercetin Q, its derivatives 1-15 and trolox T determined by measuring radical scavenging activity (DPPH) and antioxidant reducing power (FRAP).

No.	Compound	IC_50_ ± SD	
		DPPH	FRAP
**1**	diquercetin	58.8±6.2	103.1±6.2
**2**	monochloropivaloyl quercetin	27.0±0.2	12.5±0.3
**3**	tri(monochloropivalyol) quercetin	>200	>200
**4**	5-morfolinohydroxypropoxy quercetin	>200	>200
**5**	chloronaphtoquinone quercetin	28.4±3.0	64.5±0.6
**6**	pentaacetyl quercetin	>200	>200
**7**	tri(diprenylcaffeoyl) quercetin	>200	>200
**8**	di(diprenylcaffeoyl) quercetin	160.9±13.2	>200
**9**	di(tetraacetylquinoyl) quercetin	63.7±2.6	112.5±7.3
**10**	tri(trimethylgalloyl) quercetin	>200	>200
**11**	tri(diacetylcaffeoyl) quercetin	35.4±2.7	>200
**12**	tetra(acetylsalicyloyl) quercetin	>200	>200
**13**	tri(acetylferuloyl) quercetin	>200	>200
**14**	di(prenylferuloyl) quercetin	82.8±3.3	183.3±13.5
**15**	monoacetylferuloyl quercetin	>200	>200
**Q**	quercetin	16.2±1.1	12.5±0.9
**T**	trolox	34.7±1.0	39.5±2.1

Values are in µM, mean of five experimental samples ± standard deviation.

Of the screened samples the best ability to bind free radical DPPH? were monochloropivaloyl quercetin **2** (IC_50_=27 µM), chloronaphtoquinone quercetin **5** (IC_50_=28.4 µM) and tri(diacetylcaffeoyl) quercetin **11** (IC_50_=35.4 µM), which were as effective as trolox and about one half less effective than quercetin. A certain activity was determined for diquercetin **1** (IC_50_=58.8 µM), di(tetraacetylquinoyl) quercetin **9** (IC_50_=63.7 µM), di(prenylferuloyl) quercetin **14** (IC_50_=82.8 µM) and di(diprenylcaffeoyl) quercetin **8** (IC_50_=160.9 µM). The derivatives **3, 4, 6, 7, 10, 12, 13, 15** were inactive at the concentrations tested. Their IC_50_ value was greater than 200 µM.

In the second antioxidant test ferric reducing ability (FRAP) of the samples was evaluated. From Table 2 it is evident that the best values were achieved for monochloropivaloyl quercetin **2** (IC_50_=12.5 µM), which was as effective as quercetin and three times more effective than the standard antioxidant trolox. Certain activity was found for chloronaphtoquinone quercetin **5** (IC_50_=64.5 µM), diquercetin **1** (IC_50_=103.1 µM), di(tetraacetylquinoyl) quercetin **9** (IC_50_=112.5 µM) and di(prenylferuloyl) quercetin **14** (IC_50_=183.3 µM). The other compounds tested were inactive up to 200 µM concentration (**3, 4, 6–8, 10–13, 15**).

Table 3 shows the values of 50% cell growth inhibitory concentrations (IC_50_) for quercetin and its derivatives on HeLa and NIH-3T3 cells. The values were obtained from the MTT test. The highest cytotoxic effect on human cancer cell line HeLa was induced by chloronaphtoquinone quercetin **5** (IC_50=_13.2 µM), tri(diacetylcaffeoyl)quercetin **11** (IC_50_=16.5 µM), di(tetraacetylquinoyl) quercetin **9** (IC_50_=19.5 µM) and pentaacetyl quercetin **6** (IC_50_=29.6 µM). The cytotoxic effect of these derivatives on cancer cells was higher than the cytotoxicity of quercetin. Certain cytotoxicity was observed in monochloropivaloyl quercetin **2** (IC_50_=43.4 µM) and diquercetin **1** (IC_50_=64.0 µM). Eight derivatives tested were inactive, IC_50_ values were greater than 50 µM for the derivatives **7**, **8**, **10**, **12–15** and IC_50_ was greater than 100 µM for derivative **4**.

**Table 3:** Evaluation of IC50 values for quercetin Q and its derivatives 1-15 determined by MTT assay on human cervical cancer cells HeLa and non-cancer murine fibroblast cells NIH-3T3 (time of drug exposure 72 h).

No.	Compound	IC_50_ ± SD	
		HeLa cells	NIH-3T3 cells
**1**	diquercetin	64.0±7.5	32.9±1.1
**2**	monochloropivaloyl quercetin	43.4±2.8	26.8±0.5
**3**	tri(monochloropivalyol) quercetin	65.0±4.5	20.3±1.9
**4**	5-morfolinohydroxypropoxy quercetin	>100	>100
**5**	chloronaphtoquinone quercetin	13.2±1.6	16.5±0.1
**6**	pentaacetyl quercetin	29.6±1.9	15.5±0.7
**7**	tri(diprenylcaffeoyl) quercetin	>50	>50
**8**	di(diprenylcaffeoyl) quercetin	>50	>50
**9**	di(tetraacetylquinoyl) quercetin	19.5±0.8	16.1±0.4
**10**	tri(trimethylgalloyl) quercetin	>50	>50
**11**	tri(diacetylcaffeoyl) quercetin	16.5±1.5	10.6±0.1
**12**	tetra(acetylsalicyloyl) quercetin	>50	>50
**13**	tri(acetylferuloyl) quercetin	>50	>50
**14**	di(prenylferuloyl) quercetin	>50	>50
**15**	monoacetylferuloyl quercetin	>50	>50
**Q**	quercetin	35.5±1.1	20.9±0.9
**T**	trolox	34.7±1.0	39.5±2.1

Values are in µM, mean of five experimental samples ± standard deviation.

The highest cytotoxicity on the non-cancer cell line NIH-3T3 was initiated by tri(diacetylcaffeoyl) quercetin **11** (IC_50_=10.6 µM), pentaacetyl quercetin **6** (IC_50_=15.5 µM), di(tetraacetylquinoyl) quercetin **9** (IC_50_=16.1 µM) and chloronaphtoquinone quercetin **5** (IC_50_=16.5 µM). Certain cytotoxicity was observed in tri(monochloropivalyol) quercetin **3** (IC_50_=20.3 µM), monochloropivaloyl quercetin **2** (IC_50_=26.8 µM) and diquercetin **1** (IC_50_=32.9 µM). Derivatives **4**, **7**, **8**, **10**–**14** and the derivative **15** were inactive, IC_50_ values were greater than 100 and 50 µM, respectively.

**Figure 1:**
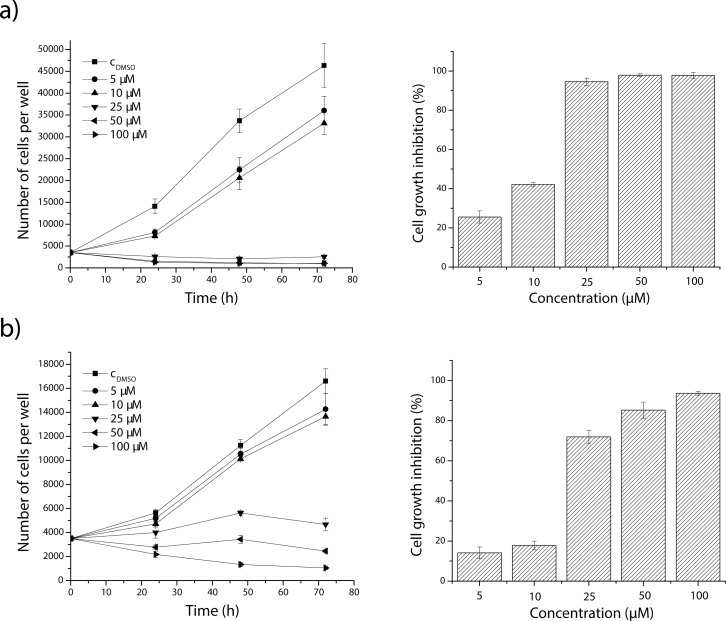
Effect of chloronaphtoquinone quercetin **5** on cell proliferation and growth inhibition of HeLa (a) and NIH-3T3 (b) cells after 72 h of exposure. C_DMSO_ – control cells tretated with 0.5% DMSO. Each point represents the mean ± SD of the six experiments.

As seen from growth curves (Figure 1 left), addition of the most effective derivative chloronaphtoquinone quercetin **5** to the medium reduced HeLa and NIH-3T3 viable cell numbers. The three highest concentrations tested (100, 50 and 25 µM) had an acute effect manifested by cell degeneration (NIH-3T3) or total inhibition of cell proliferation (HeLa) after 72 h of treatment. The cytotoxic effect of chloronaphtoquinone quercetin **5** at concentrations 5 and 10 µM was directly proportional to the concentration and time of influence. Inhibition of cell proliferation was 94.54% and 71.88% (for 25 µM), 97.89% and 85.24% (for 50 µM) and 97.72% and 93.66% (for 100 µM) for HeLa and NIH-3T3 cells, respectively (Figure 1 right).

**Figure 2:**
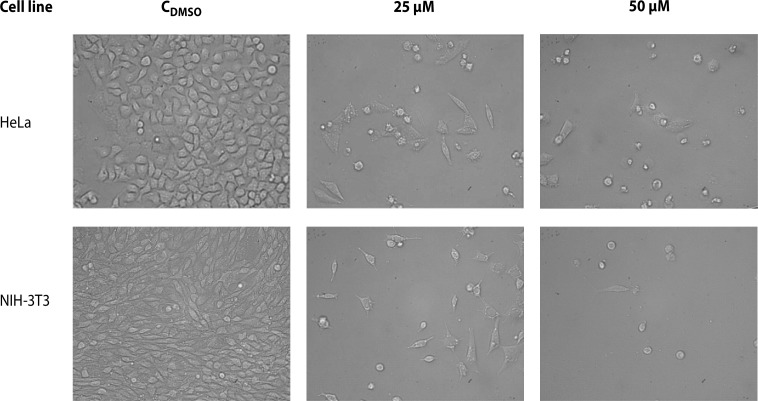
Effect of chloronaphtoquinone quercetin **5** in final concentrations of 25 and 50 µM on morphology of HeLa and NIH-3T3 cells during 72 h of cultivation. C_DMSO_ – control cells treated with 0.5% DMSO. Magnification 200×.

The cytotoxic action of the effective chloronaphtoquinone quercetin **5** was further observed also by light microscopy after 72 h of HeLa and NIH-3T3 cells cultivation. Microscopic observations (Figure 2) showed that control cells grew on the surface of the cultivation vessels and during 72 h of incubation a monolayer was formed. On the other hand, a significant decrease in cell number was found in cells treated with both concentrations studied (25 µM and 50 µM). Derivative **5** induced morphologic changes of HeLa and NIH-3T3 cells, which resulted within 72 h in cell rounding and cell separation from the cultivation surface.

## Discussion

Many health beneficial effects of flavonoids are attributed to their ability to act as antioxidants. Experiments showed that flavonoids as single electron donors can stabilize and eliminate many radical forms, which in conditions of oxidative stress may initiate mutagenesis or carcinogenesis. The chemical structure of flavonoids allows them to chelate transition metals (Miller, 1996). The activity of antioxidants such as flavonoids may prevent damage of DNA, proteins, lipids and other important biomolecules.

Quercetin is an effective antioxidant among natural compounds (Baghel *et al.*, 2012). Our testing showed that in binding free radicals and in reducing metals it exerts better activities than the standard antioxidant trolox. Novel acylated and conjugated quercetin derivatives displayed lowered antioxidant capacity in comparison with unmodified quercetin (except monochloropivaloyl quercetin **2** in the FRAP method) (Table 2). Of the quercetin derivatives screened the most active in both tests were monochloropivaloyl quercetin **2** and chloronaphtoquinone quercetin **5**.

Investigations of lowered antioxidant capacity of compounds upon quercetin structure modification are in accordance with the literature because free hydroxyl groups in the structure are necessary to scavenge free radicals or chelate metals. Salem *et al.* (2011) found that acylation of isorhamnetin-3-O-glucoside decreased its radical scavenging activity. Lue *et al.* (2010) reported lowered ability of rutin laurate and rutin palmitate to bind metals. Tert-butylhydroxylation of quercetin lowered its ability to scavenge free radicals (Lebeau *et al.*, 2000). Yet it seems that antioxidant activity in lipophilic media was improved in many cases after structure lipophilization (Filipe *et al.*, 2009; Viskupicova *et al.*, 2010). Since quercetin derivatives are metabolized to quercetin in the human body (Materska, 2008), reduced antioxidant capacity in the case of derivatives should not mean a serious problem.

In the last decade, there has been growing interest in searching for cancer prophylactic and therapeutic effects of natural compounds. Many *in vitro* and some *in vivo* studies confirmed that flavonoids displayed a range of anticancer actions. They are able to inhibit carcinogen activation, cancer cell proliferation, angiogenesis and metastasis. These compounds also induce apoptosis, have antioxidant and anti-inflammatory effects and reverse multidrug resistance (Genoux *et al.*, 2011).

We monitored prepared quercetin derivatives for their cytotoxic action on human cervical cancer cells HeLa in the concentration range from 5 µM to 100 µM. For comparison non-cancer murine fibroblast cells NIH-3T3 were also evaluated. The MTT test and light microscopy were used for cytotoxicity measurements.

The cytotoxic screening of quercetin derivatives on HeLa and NIH-3T3 cells demonstrated that chloronaphtoquinone quercetin **5**, tri(diacetylcaffeoyl)quercetin **11**, di(tetraacetylquinoyl) quercetin **9** and pentaacetyl quercetin **6** were more effective than quercetin. The comparison of the structures of quercetin derivatives and their cytotoxic effect showed that acetylated esters were the most effective derivatives.

Other authors published similar results. Acylation of isorhamnetin-3-O-glucoside with ethyl laurate and ethyl butyrate increased their antiproliferative activity against Caco-2 cells (Salem *et al.*, 2011). Cárdenas *et al.* (2006) found that naphtoflavons were slightly better in antiproliferation than initial flavone molecules. Acylation and alkylation of epicatechin resulted in enhanced inhibitory activity on cancer cells (Park *et al.*, 2004). Introduction of various acyl and alkyl moieties to the flavonoid skeleton caused improvement of its cytotoxic properties. The most effective were dimethoxy flavonoids (Ou *et al.*, 2011). In our experiments, acetyl moieties probably helped quercetin to penetrate through the cell membranes into cancer cells and thus these derivatives become more effective.

As seen in Figure 1, the cytotoxic effect of chloronaphtoquinone quercetin **5**, the most effective derivative, varied with concentration and time of influence. The three highest concentrations tested induced acute cytolytic effects, which were demonstrated by total degeneration of HeLa cells after 72 h of treatment. Lower concentrations induced toxicity that was concentration- and time-dependent. On the other hand, acute cytolytic effect on non-cancer NIH-3T3 cells was induced only by derivative concentration of 100 µM. The concentration of 50 µM caused total cell inhibition after 72 h of exposures. Lower derivative concentrations evoked toxicity that was concentration- and time-dependent.

Light microscopy revealed that both concentrations studied of chloronaphtoquinone quercetin **5** (25 and 50 µM) induced morphologic changes which resulted within 72 h in cell rounding and cell separation from the cultivation surface.

According to the obtained data we can conclude also that the antioxidant activity was not in correlation with the cytotoxic action. Although structure modification lowered the antioxidant properties of quercetin, cancer cell inhibitory activities were enhanced mainly in the case of compound acetylation.

The best effects were found for chloronaphtoquinone quercetin. This compound inhibited the growth of non-cancer and cancer cells at comparable level (IC_50_=13.2 µM for HeLa cells, IC_50_=16.5 µM for NIH-3T3 cells).
